# Radix puerariae extracts ameliorate paraquat-induced pulmonary fibrosis by attenuating follistatin-like 1 and nuclear factor erythroid 2p45-related factor-2 signalling pathways through downregulation of miRNA-21 expression

**DOI:** 10.1186/s12906-016-0991-6

**Published:** 2016-01-12

**Authors:** Ming-wei Liu, Rong Liu, Hai-ying Wu, Yi-yun Li, Mei-xian Su, Min-na Dong, Wei Zhang, Chuan-yun Qian

**Affiliations:** 1 Department of Emergency, the First Affiliated Hospital of Kunming Medical University, 295 Xichang Road, Wu Hua District, Kunming, 650032 China; 2 Emergency Intensive Care Unit, the Second Affiliated Hospital of Kunming Medical University, Kunming, 650106 China

**Keywords:** Lung fibrosis, Radix puerariae extracts, miR-21, Connective tissue growth factor, Nuclear factor erythroid 2p45-related factor-2, Nuclear factor-κB, Oxidative stress, Mice

## Abstract

**Background:**

Puerarin, extracted from Radix puerariae, was reported to ameliorate airway inflammation, lung injury and lung fibrosis induced by paraquat (PQ) in mice. However, effects of Radix puerariae extracts (RPEs) on lung fibrosis or signalling pathways in PQ-induced lung injury have not been well studied. Therefore, the goals of our study were to investigate whether Radix puerariae extracts are antifibrotic in a paraquat (PQ) induced lung fibrosis model in mice and to propose possible mechanisms of action of the RPE effects.

**Methods:**

We used a long-term exposure model of PQ-induced lung fibrosis in mice to evaluate effects of antioxidant-containing RPE. We examined effects of miR-21 on follistatin-like 1 (Fstl 1) pathways and oxidative stress in the lung. Gene expression levels of miR-21, Fstl 1, transforming growth factor-β1 (TGF-β1), connective tissue growth factor (CTGF), collagen-1 and collagen III were measured by real-time PCR. Protein expression levels of Fstl 1(FSTL1), heme oxygenase-1 (HO-1), nuclear factor erythroid 2p45-related factor-2 (Nrf2), Smad2/3, p38MAPK, nuclear factor-κB 65 (NF-κB65), and matrix metalloproteinase-9 were detected by western blotting. FSTL1 andalpha-smooth muscle actin (α-SMA) in lung tissue were detected by immunohistochemistry. Malondialdehyde, superoxide dismutase (SOD), reduced (GSH) and oxidised (GSSH) glutathione and reactive oxygen species levels, hydroxyproline and total lung collagen were also determined.

**Results:**

Long-term challenge with PQ enhanced miRNA-21 (miR-21), Fstl 1 pathways, oxidative stress and development of fibrotic features in the lungs. RPE reduced features of lung fibrosis by blocking Fstl 1 pathways and oxidative stress through decreased miR-21 expression. This was accompanied by suppression of CTGF, TGF-β1, vascular endothelial growth factor, collagen I, and collagen III. In addition, PQ-induced activation of NF-κB, Nrf2 and α-SMA were enhanced by puerarin. We also found that puerarin increased HO-1, SOD and GSH levels.

**Conclusions:**

These findings demonstrated that RPEs blocked PQ-induced Fstl 1 pathways and oxidative stress by inhibiting miR-21 expression, leading to attenuation of PQ-induced lung fibrosis.

## Background

Paraquat, (PQ) widely used as an herbicide, is controversial because of the high mortality of PQ exposure, with a typical case fatality of 50–90 % [1]. The major cause of death by PQ poisoning is respiratory failure, a consequence of oxidative injury to the alveolar epithelium [2]. The primary organ system affected by PQ toxicity is the lung, where PQ is accumulated via an active transport process into Clara cells and alveolar type I and type II epithelial cells. Its initial effects are pulmonary oedema, infiltration of inflammatory cells and damage to the alveolar epithelium, followed by the resulting lung fibrosis and respiratory failure [2–4]. The high fatality of PQ is caused by its inherent toxicity and the lack of any effective treatment [2]. Overall mortality remains above 50 % even in intensive care facilities [2].

The traditional Chinese herbal medicine Radix puerariae has been used to treat alcoholism for thousands of years [5, 6]. In a recent report, puerarin, extracted from Radix puerariae, had antioxidant and antithrombotic effects and decreased cell injuries caused by lipid peroxidation by stabilising cell membranes [7–9]. Such findings indicated that puerarin might prevent PQ-induced pulmonary fibrosis.

MicroRNAs (miRNAs) are a class of non-coding small RNAs, 18–22 nucleotides in length, which bind to target genes and suppress their translation and/or induce degradation of the target gene mRNA. miRNA-21 (miR-21) is one of the most important miRNAs upregulated during fibrogenesis in various tissues [10]. TGF-β1 canstimulate miR-21 expression, detected in the liver, heart, kidneys and lungs of mice [10]. miR-21 is upregulated in the lungs of patients with idiopathic pulmonary fibrosis (IPF) and inhibition of miR-21 attenuates lung fibrosis in mice [11, 12].

Follistatin-like 1 (FSTL1, coded by the Fstl 1 gene), an extracellular glycoprotein, was originally cloned from an osteoblastic cell line as a TGF-βl inducible gene [13]. FSTLl is highly homologous to follistatin, which is effective against acute lung injury and bleomycin-induced lung fibrosis by blocking activin and TGF-β [14]. The physiological function of FSTL1 remains unclear.

TGF-β1 is known to enhance fibrosis by increasing fibroblast growth and collagen deposition and promoting differentiation of fibroblasts into myofibroblasts [15], which secrete collagen and other extracellular matrix components. CTGF acts as a cofactor with TGF-β1 to induce fibroblasts to become myofibroblasts, leading to collagen deposition and ultimately resulting in organ scarring and fibrosis [16].

Oxidative stress is one important molecular mechanism underlying fibrosis in a variety of organs, including the lungs [17]. The lungs and all other organs express a wide variety of antioxidants to protect them against oxidative stress. These include catalase, GSH, SOD and HO-1. In addition, Nrf2 is a transcription factor regarded as being the “master regulator” of the antioxidant response [17].

In this study, we demonstrated that antioxidants present in a Radix puerariae extract (RPE) could alter pulmonary fibrosis. We also further explored the underlying signalling pathways of these anti-fibrotic effects using long-term PQ exposure in mice as a model system. We found that RPE ameliorated lung fibrosis and blocked reactive oxygen species (ROS)-associated Fstl 1 activation, oxidative stress and inflammatory signalling pathways by downregulating miR-21 expression.

## Methods

### Preparation of RPE

Radix puerariae, the root of Pueraria lobata (Willd) Ohwi (Shanghai Leiyunshang Pharmaceutical Co. Ltd., Shanghai, China) was ground, extracted three times with 70 % alcohol for 2 h, concentrated with a vacuum rotary evaporator and freeze-dried. The dried powder was then dissolved in distilled water and filtered. It was chromatographed on a macroporous resin D101 column (11.5 cm × 85.5 cm) eluted with distilled water followed by 70 % ethanol. The 70 % ethanol eluate was dried with a rotary evaporator and stored in the dark at 4 °C. A yield of 3.89 g RPE was obtained from 100 g of dried Radix puerariae.

### Extraction and analysis of Radix puerariae

The dried powder sample (200 mg) of RPE was extracted with 5 mL methanol for 12 h on a test tube rotator and centrifuged at 2000 × g for 10 min and the supernatant collected and evaporated in a Speed-Vac sample concentrator (model SPD 111 V, Thermo Savant, LaJolla, CA, USA). For HPLC analysis, extracts were redissolved in HPLC grade methanol and filtered through a nylon filter (0.45 μm, National, Salt Lake City, UT, USA). Sample injection volume for HPLC was 20 μL. The HPLC system was equipped with a pump (L2130; Hitachi, Tokyo, Japan), auto sampler (L-2200; Hitachi) and a UV detector (L-2400; Hitachi) controlled with “Lachrome Elite” software (Waters 2487 instrument software).

HPLC separation was performed on a 250 mm × 4 mm C18 reversed-phase column (5 μm, LichroCART; Merck KGaA, Darmstadt, Germany) protected by a guard column of the same material. The HPLC method was as described by Goyal and Ramawat [8] with small modifications. The solvent system was composed of solvent A (0.0025 % trifluoroacetic acid in water) and solvent B (80 % acetonitrile (E. Merck, Mumbai, India) in solvent A). The column was eluted with successive gradients of solvent A and solvent B, with the percent solvent A programmed as follows: 0–2 min, 85 %; 2–5 min, 85 % → 80 %; 5–15 min, 80 % → 50 %; 15–20 min, 50 % → 40 %; 20–30 min, 40 % → 30 %; 30–35 min, 30 % → 20 %; 35–45 min, 20 % → 0 %; 45–48 min, 0 %; 48–50 min, 0 % → 85 %; 50–55 min, 85 %. Separation was performed at a flow rate of 1.0 mL/min and chromatographic peaks at 254 nm were monitored. The compounds were quantified from a calibration curve constructed with standard solutions. To analyse components of RPE, the concentrations of the chemical reference substance standards used for plotting the calibration curve ranged from 1.0 to 5.0 μg. Each HPLC run was repeated three times.

**Fig. 1:**
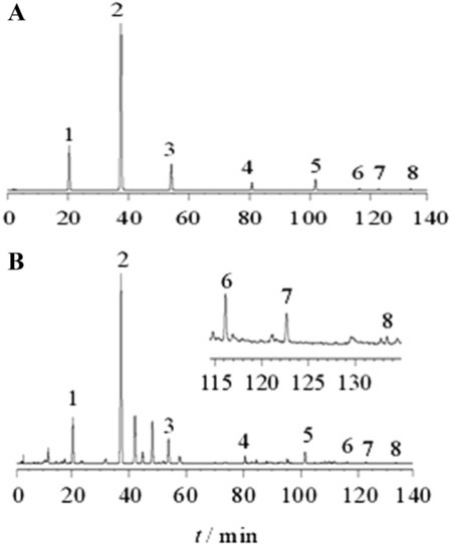
Chromatogram of the chemical reference substances and sample. **a** Chromatogram of the chemical reference substances. **b** Chromatogram of various samples: 1, hydroxypuerarin; 2, puerarin; 3, daidzin; 4, genistin; 5, daidzein; 6, genistein; 7, formomonetin; 8, biochanin A

The HPLC analysis indicated that 100 mg RPE contained 49.4 mg puerarin, 1.3 mg daidzin, and 0.16 mg daidzein, indicating that puerarin was its major component (Fig. 1).

### Animals

C57BL/6J (B6) mice were purchased from Kunming Medical University Laboratory Animal Center (Kunming, China). All mice were housed in the Animal Care Facility of Kunming Medical University and maintained in a pathogen-free environment. The mice (8–9 week old and weighing 20–30 g) used in the experiment were housed in a vivarium maintained at 23 °C with a 12:12 h light/dark cycle (lights off at 7.00 p.m.). They received a standard laboratory diet and water *ad libitum*. All experiments were approved by the Ethics Committee of Kunming Medical University (Yunnan, China) (Approval number: TCM-2011-041-E17) and performed according to The Guidelines of the Animal Care Committee of Kunming Medical University.

### In vivo miR-21 knockdown using locked nucleic acid-modified anti-miR-21

Locked nucleic acid (LNA)-modified scrambled or anti-miR-21 oligonucleotides (Exiqon, Woburn, MA, USA) were diluted in saline (5 mg/mL) for administration through intraperitoneal (i.p.) injection (10 mg/kg) at least 30 min before PQ exposure [18].

### Reagents

PQ aqueous solution (active ingredient content of 200 g/L, product license number: XK13-003-00058) was from Chuandong Agrochemical Co., Ltd, (Guangdong, China). An enhanced chemiluminescence (ECL) kit was from Perkin Elmer Life Sciences, Inc. (Boston, MA, USA). SYBR fluorescence quantitative reverse transcription polymerase chain reaction (PCR) kit was from Takara Company (Tokyo, Japan). TRIzol reagent was from Invitrogen Corporation (Carlsbad, CA, USA), Mouse TGF-β1 and MMP-9 ELISA kits were from Bender Medsystem (Vienna, Austria).

### Animal treatments

In the first experiment, mice were weighed and randomly divided into five groups (five mice per group) to assess protective effects of puerarin on pulmonary fibrosis. For induction of pulmonary fibrosis, PQ (10 mg/kg) or saline, as a control, was injected i.p. into mice [3]. Group 1 was untreated (or, where indicated, received only saline) and served as the control group; Group 2 received PQ (10 mg/kg) to induce pulmonary fibrosis and served as the “model group”; Group 3 received PQ to induce pulmonary fibrosis and was also treated with RPE at a dose of 30 mg/kg i.p. once per day. On day 14 after PQ injection, mice were killed and lungs harvested. A small portion of each lung was fixed with 10 % formalin and embedded in paraffin for haematoxylin-eosin (HE) and Masson’s trichrome staining.

### Real-time PCR analysis

For RNA isolation, lung tissues were frozen in liquid nitrogen and stored in a −80 °C freezer until use. Total RNA was extracted from frozen lung tissue (left lung) with TRIzol reagent and amplified with a PCR single-step kit (Promega, Madison, WI, USA) according to the manufacturer’s instructions. Real-time-PCR was performed with a PTC-200 DNA Engine PCR cycler (Bio-Rad Laboratories, Inc., Hercules, CA, USA). The primers (Table 1) were designed based on published sequences of these genes and synthesised by Invitrogen [19, 20]. PCR was performed in a total volume of 20 μL containing 20 mM Tris–HCl, 50 mM KCl, 1.25 mM MgCl2, 0.2 mM dNTP, 0.5 mMprimer, 1 U TaqDNA polymerase and 0.5 μL cDNA. Cycle parameters were as follows: 95 °C for 3 min, 25 cycles (98 °C for 30 s, 60 °C for 40 s, and 72 °C for 60 s) and 72 °C for 5 min. Ordinary PCR products were separated on a 2 % agarose gel. β2-Actin was used as an endogenous controland values for each sample were normalised according to its β2-actin content. The mRNA expression levels of target genes were calculated using the 2^-ΔΔCt^ method [21, 22].

**Table 1:** Primer sequences for the genes to validate microarray analysis by real-time-PCR

miRNA-21	F: 5′-TGACATCGCATGGCTGTA-3′	336 bp
	R:5′-GATGCTGGGTAATGTTTGAAT-3′	
Fstl1	F: 5′-TTATGATGGGCACrGCAAAGAA-3′	405 bp
	R: 5′- ACTGCCTTTAGAGAACCAGCC-3′	
TGF-β1	F: 5′-CAAGAACAAGGCAGACTTATCGC-3′	418 bp
	R: 5′-TCTGATTATCTCGCACCAGGAAG-3	
CTGF	F: 5′-TTCGGTGGTACGGTGTACCGCA-3′	359 bp
	R: 5′-ACGAACGTCCATGCTGCACAGG-3′	
collagen-1	F: 5′-ACCTGCGTACAGAACGGCCT-3′	318 bp
	R: 5′-ACAACACCTTGCCGTTGTCGC-3′	
Collagen III	F: 5′-GGATCTGTCCTTTGCGATGAC-3′	276 bp
	R: 5′-GCTGTGGGCATATTGCACAA-3′	
β-actin	F: 5′-CCTCATGAAGATCCTGACCG-3′	190 bp
	R: 5′-ACCGCTCATTGCCGATAGTG-3′	

### Western blotting

Lung homogenates were prepared in lysis buffer (50 mM Tris–HCl, 150 mM NaCl, 1 % NP-40, 0.5 % sodium deoxycholate, 2 mM NaF, 2 mM EDTA, 0.1 % SDS and a protease inhibitor cocktail tablet (Roche Applied Science, Indianapolis, IN, USA). Protein concentrations were quantified by the BCA method (Pierce Biotechnology, Inc., Rockford, IL, USA). An equal amount of protein (30 mg) from each sample was loaded on respective gel lanes. Samples and pre-stained molecular weight markers (Bio-Rad) were electrophoresed on 12 % Tris-glycine polyacrylamide gels, then protein bands were electrophoretically transferred onto polyvinylidene difluoride (PVDF) membranes (Millipore Corp., Marlborough, MA, USA). Membranes were blocked for 1 h at room temperature with 5 % bovine serum albumin (BSA) and then incubated overnight at 4 °C with the following primary antibodies: anti-MMP-9 (Santa Cruz Biotechnology, Dallas, TX, USA), anti-p-p38MAPK (Bio-Rad Laboratories Inc.), anti-NF-kB65 (DAKOCorp, Carpinteria, CA, USA), anti-HO-1 (Cell Signaling, Beverly, MA, USA), anti-Nrf2 (Cell Signaling), anti-FSTL1 (R&D systems, Minneapolis, MN, USA), anti-p-Smad2/3 (Serotec Ltd, Oxford, United Kingdom), anti-TGF-β1 (Sigma, St. Louis, MO, USA), anti-CTGF (Santa Cruz Biotechnology), anti-collagen III (Santa Cruz Biotechnology), anti-collagen-1 (Invitrogen), and anti-β-actin (Sigma-Aldrich), each diluted 1:1000 in Tris-buffered saline with Tween-20 (TBST). β-actin blotting served as the control to confirm protein loading. After washing with TBST, membranes were incubated with the corresponding horseradish peroxidase-linked anti-rabbit antibody (Pierce) diluted in TBST (1:20000) for 1 h at room temperature. After further washing with TBST, immunoreactive bands were visualised by enhanced chemiluminescence (ECL) and quantified by densitometry using Bio-Rad Universal Hood and Quantity One software (Bio-Rad). Results were normalised to β-actin levels in the same lanes.

### Enzyme-linked immunosorbent assay (ELISA)

At 24 h after the last challenge, bronchoalveolar lavage (BAL) fluid was obtained from the anaesthetised mice with 1 mL sterile isotonic saline. Lavage was performed four times and the total volumes pooled for each mouse. Lavage fluid samples were immediately centrifuged at 2000 × g for 10 min at room temperature and stored at −80 °C until use. TGF-β1 and MMP-9 levels were then assayed with TGF-β1 and MMP-9 ELISA kits according to the manufacturers’ instructions.

### Immunohistochemistry

Immunostaining was performed on lung sections after antigen retrieval using Retrievagen A (Zymed, South San Francisco, CA, USA) at 100 °C for 20 min and then quenching endogenous peroxidases with 3 % H_2_O_2_. Sections were blocked with 2 % BSA in PBS followed by staining with primary anti-FSTL1 and α-SMA at room temperature for 1 h. Sections were then washed. After application of the secondary antibody (Sigma-Aldrich), tissue staining was developed with Vectastain ABC (Vector Labs, Burlingame, CA, USA) and 3,3′-diaminobenzidine (Vector Labs). With Image Pro Plus image analysis software (Media Cybernetics, Bethesda, MD, USA), FSTL1 and α-SMA positive staining in lung tissue were determined and each staining intensity was expressed as positive units.

### Total lung collagen

Total lung collagen was determined by measuring total soluble collagen (Sircol Collagen Assay, Biocolor, Belfast, Northern Ireland). The left lung was homogenised in 5 mL 0.5 M acetic acid containing pepsin (1 mg/10 mg tissue; Sigma-Aldrich) and incubated (24 h, 24 °C, with mixing at 240 rpm). Sircol dye was added (1 mL/100 mL, with mixing for 30 min) and then the sample was centrifuged (12,000 × g for 12 min). The pellet was resuspended in 1 mL 0.5 M NaOH. The optical density at 540 nm was measured with a spectrophotometer.

### Measurement of intracellular ROS

ROS were measured as previously described [23]. BAL cells were washed with PBS and incubated for 10 min at room temperature with PBS containing 3.3 μM 2′,7′-dichlorofluorescein (DCF) diacetate (Molecular Probes, Eugene, OR, USA) to label intracellular ROS. DCF stained cells were analysed by fluorescence-activated cell sorting (1 × 10^4^ cells).

### SOD activity assay

SOD activity was estimated as described by Kakar et al. [24]. The reaction mixture contained 0.1 mL phenazine methosulphate (186 μM) and 1.2 mL sodium pyrophosphate buffer (0.052 mmol, pH 7.0). Lung homogenate was prepared in 10 mL ice-coldlysis buffer (50 mM phosphate buffer with 1 m Methylene diamine tetraacetic acid (EDTA) per g tissue and centrifuged twice (once at 1500 × g for 10 min, and the supernatant centrifuged again at 10,000 × g for 15 min). An aliquot (0.3 mL) of the resulting supernatant was added to the reaction mixture. The enzymatic reaction was initiated by adding 0.2 mL NADH (780 μmol) and stopped after 1 min by adding 1 mL glacial acetic acid. The amount of chromogen formed was measured by absorbance at 560 nm. Results are expressed in units/mg protein.

### Measurement of malondialdehyde (MDA)

Lung tissue homogenates from control and experimental groups were prepared in 0.1 M Tris–HCl buffer (pH 7.4) at 4 °C. The resulting tissue homogenates were used for all biochemical measurements unless otherwise indicated. MDA content was determined by a colorimetric assay with a commercially available kit (Jiancheng Bioengineering Institute, Nanjing, China) according to the manufacturer’s instructions. Briefly, MDA content in the lung was measured according to the thiobarbituric acid method based on the formation of a red complex when MDA reacted with thiobarbituric acid. The absorbance was spectrophotometrically measured at 532 nm.

### Measurement of GSH and GSSG in lung tissues

Lung tissues were homogenised with 10 mL ice-cold buffer (50 mM phosphate buffer containing 1 mM EDTA per gram tissue). After centrifugation at 10,000 × g for 15 min at 4 °C, the supernatants were removed, deproteinated with 7.5 μl 5 M KOH per 500 μl volume and then stored at −20 °C until assayed. Total GSH and GSSG levels were determined with a GSH Assay Kit (Cayman Chemical Company, Ann Arbor, MI, USA) according to the manufacturer’s protocol.

### Hydroxyproline assay

Collagen content was determined based on hydroxyproline (HYP) levels [25]. Briefly, 10 mg lung tissue was minced in 1 mL 6 M HCl, hydrolysed and then incubated overnight at 120 °C. Five milliliters 0.5 M acetic acid was added and the pH adjusted to between 6.0 and 6.5 with 0.2 M NaOH. Chloramine T solution (1 mL, 0.05 M) was added and the mixture was incubated for 20 min at room temperature. Aldehyde-perchloric acid (1 mL of a 0.5 M solution) was added and the mixture incubated at 60 °C for 15 min. Absorbance was then recorded at 550 nm. Results are expressed as μg/mg wet lung weight with HYP values read from a standard curve.

### Histopathology

Whole lungs were inflated *in situ* with PBS-buffered formalin (4.5 %, pH 7.0) (Roth, Darmstadt, Germany) and then carefully immersed in more PBS-buffered formalin. Lung tissue samples were paraffin-embedded and 5-μm-thick sections were prepared and stained with haematoxylin/eosin (H/E) and Masson’s or Elastica-van-Gieson staining. The degree of lung fibrosis was assessed in lung samples as described by Ashcroft and colleagues [26] with a numerical scaling system. In the scaling system, 0 indicates normal lung and eight indicates total fibrous obliteration of the field. Mean lung fibrosis degree, as defined by Ashcroft et al. [26], was calculated from individual scores of~15 microscopic fields analysed per mouse lung.

### Statistical analysis

All values were expressed as the means ± standard deviation (SD) or means ± standard error of the mean (SEM), as indicated in the individual figure legends. A one-way ANOVA followed by the Student-Newman-Keuls test was used to compare the differences among multiple groups. Pulmonary fibrosis scores were compared using nonparametric methods. The significant level was defined by a *P* value of 0.05. The SPSS 13.0 software package (SPSS, Inc., Chicago, IL, USA) was used for statistical analyses.

## Results

### Effect of miR-21 knockdown on miR-21 expression in lungs from PQ-treated mice

**Fig. 2:**
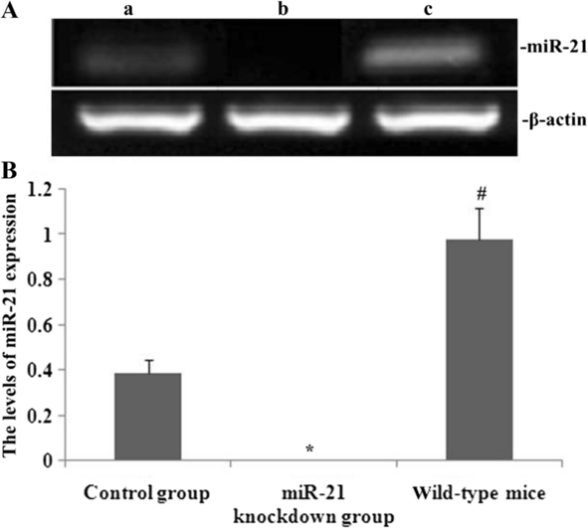
Effect of miR-21 knockdown on miR-21 expression in lungs from PQ-treated mice. Groups of mice were treated with PQ for 14 days to induce pulmonary fibrosis.miR-21 expression in pulmonary tissue was determined using real-time PCR. **a** Typical patterns of miR-21 expression as assessed by real-time PCR (a, control (scrambled oligonucleotide) group; b, miR-21 knockdown group; c, wild type group); **b** Statistical analysis of miR-21 expression. Data are means ± SD from triplicate experiments. *P < 0.05 vs control group; #*P* < 0.05 vs miR-21 knockdown group

We investigated effects of miR-21 knockdown on miR-21 expression in lung tissue from mice treated with PQ. Groups of mice were treated with PQ for 14 d to induce pulmonary fibrosis. This pulmonary fibrosis model was established in both miR-21 knockdown and wild-type (WT) mice. miR-21 expression in pulmonary tissue was determined by real-time PCR. As shown in Fig. 2, real-time PCR demonstrated no miR-21 expression in lungs from the miR-21 knockdown mice, that is, those that had been treated with the anti-miR-21 oligonucleotides.

### miR-21 knockdown decreased FSTL1 expression and mitigated PQ-induced pulmonary fibrosis

**Fig. 3:**
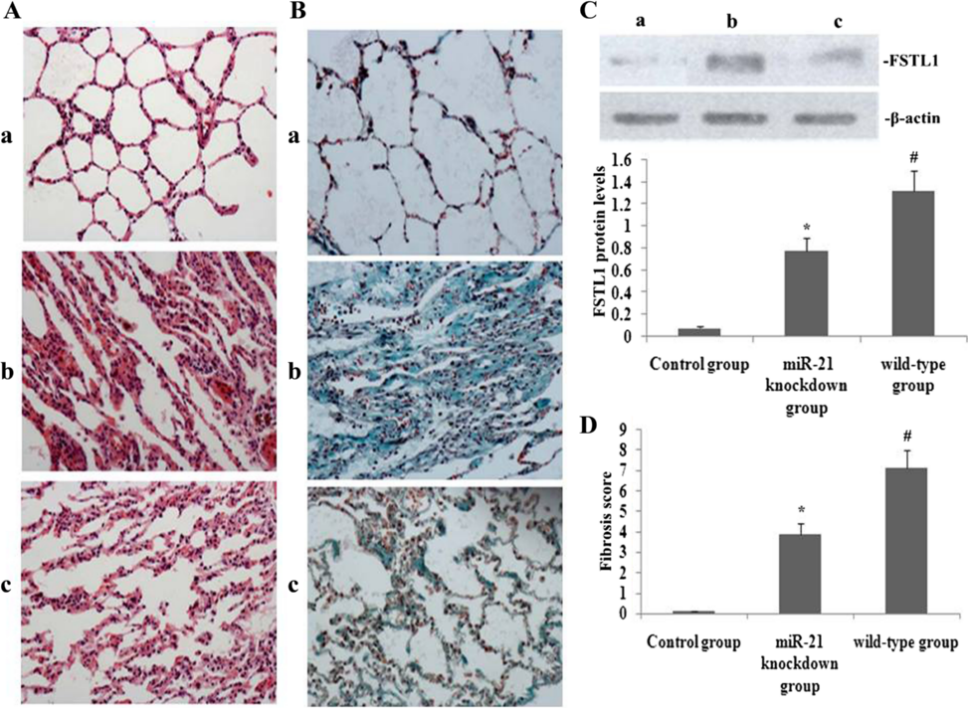
Effect of miR-21 knockdown on FSTL1 expression and PQ-induced pulmonary fibrosis. On day 14 after administration of RPE and PQ challenge, FSTL1 expression and pulmonary fibrosis were assessed. **a** and **b** Histopathological changes in lung tissues from mice (**a**: HE staining, 200× magnification; **b**: Masson’s trichrome staining, 200× magnification; a, control (scrambled oligonucleotide); b, miR-21 knockdown; c, WT); **c**: Representative western blots showing FSTL1 levels (a, control (scrambled oligonucleotide); b, miR-21 knockdown; c, WT) and statistical analysis of FSTL1 protein levels. Data are expressed as means ± SEM. *P < 0.05, vs the control group; #*P* < 0.05 vs the miR-21 knockdown group. **d** Statistical summary of pulmonary fibrosis scores in mice. Pulmonary fibrosis scores and levels of FSTL1 expression were expressed as means ± SD from triplicate experiments. *P < 0.05 vs the control group; #*P* < 0.05 vs the miR-21 knockdown group

Western blotting was performed to observe the effect of miR-21 knockdown on FSTL1 protein levels in PQ-induced pulmonary fibrosis. As shown in Fig. 3, after PQ treatment, lung expression of FSTL1 was increased and pulmonary fibrosis exacerbated in miR-21 knockdown and wild-type (WT) mice. However, protein expression of FSTL1 was significantly decreased and pulmonary fibrosis was attenuated in the miR-21 knockdown mice, as compared with in WT mice.

### RPE blocked miR-21, FSTL1, p-p38MAPK, NF-kB65, p-Smad2/3 and matrix metalloproteinase 9 (MMP-9) expression in lungs from PQ-treated mice

**Fig. 4:**
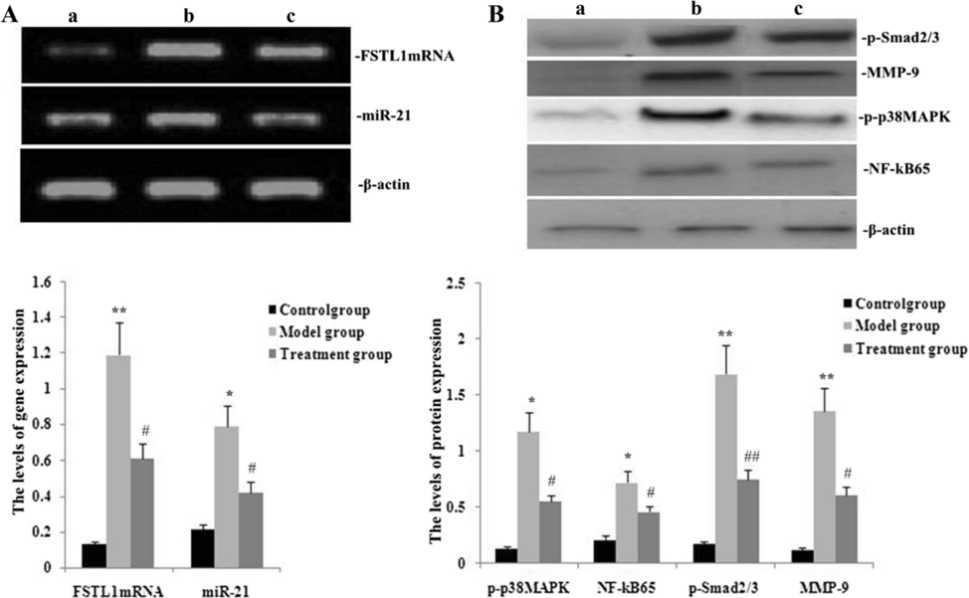
Effects of RPE on miR-21, FSTL1, p-p38MAPK, NF-kB65, p-Smad2/3, and MMP-9 expression in lungs from mice treated with PQ. On day 14 after administration of RPE and PQ challenge, miR-21, FSTL1, p-p38MAPK, NF-kB65, p-Smad2/3 and MMP-9 expression were assessed by real-time PCR (**a**) and p-p38MAPK, NF-kB65, p-Smad2/3 and MMP-9 protein levels by western blotting (**b**) Groups are as defined in Methods. (a, control; b, model group; c, treatment group). Values are means ± SD as determined from three independent experiments. *P < 0.05 and **P < 0.01 vs the control group; # *P* < 0.05 and # # *P* < 0.01 vs the model group. (The “model group” for PQ-induced lung fibrosis is defined in Methods.)

To assess effects of RPE in PQ-induced lung fibrosis, we measured miR-21 and FSTL1 gene expression with real-time PCR and p-p38MAPK, NF-kB65, p-Smad2/3 and MMP-9 protein expression with western blotting in lung tissue from mice on day 14 after PQ challenge. As shown in Fig. 4, expression levels of miR-21, FSTL1, p-p38MAPK, NF-kB65, p-Smad2/3 and MMP-9 in the lung were increased during PQ-induced pulmonary fibrosis. In mice also treated with RPE, expression of all these genes was significantly decreased.

### RPE treatment downregulated expression of TGF-β1, CTGF, collagen III and collagen I in the lung

**Fig. 5:**
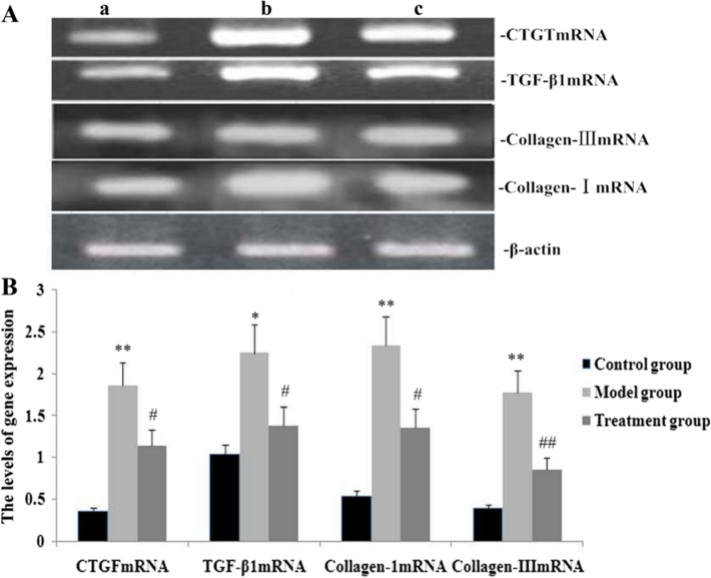
Effects of RPE treatment on gene expression of TGF-β1, CTGF, collagen III and collagen I in the lung. **a** Representative real-time PCR showing expression levels of TGF-β1, CTGF, collagen III and collagen I in mice on day 14 after the administration of RPE (a, control group; b, model group; c, treatment group). **b** Statistical summary of the 2^(−delta delta ct) analyses on mRNA expression in mice. Data are the means ± SD as determined from three independent experiments. *P < 0.05 and**P < 0.01 vs the control group; # *P* < 0.05 and # # *P* < 0.01 vs the model group

**Fig. 6:**
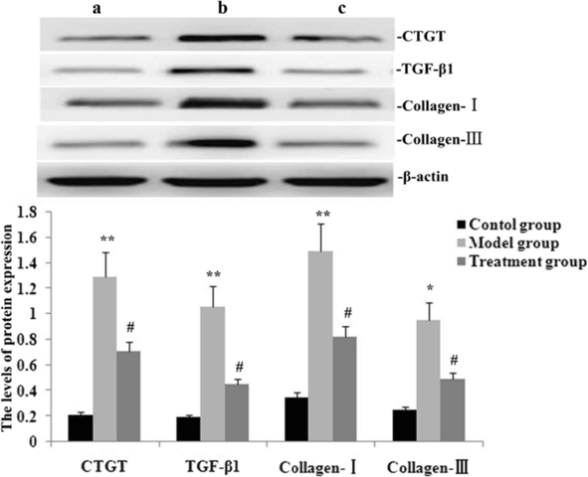
Effect of RPE treatment on protein expression of TGF-β1, CTGF, collagen III and collagen I in the lung. **a** Representative western blots showing expression levels of TGF-β1, CTGF, collagen III and collagen I in mice on day 14 after administration of RPE (a, control group; b, model group; c, treatment group). **b** Statistical summary of the densitometric analyses of protein expression in mice. Data are the means ± SD as determined from three independent experiments. *P < 0.05 and**P < 0.01 vs the control group; # *P* < 0.05 and # # *P* < 0.01 vs the model group

To clarify effects of RPE on the expression of TGF-β1, CTGF, collagen III and collagen I genes under challenge by PQ, mRNA and protein expressions of TGF-β1, CTGF, collagen III, and collagen I were measured by real-time PCR and western blotting, respectively. The mRNA and protein expression levels of TGF-β1, CTGF, collagen III and collagen I in the mouse lung were significantly increased during PQ-induced pulmonary fibrosis (*P* < 0.05, Figs. 5, 6). However, these mRNA (Fig. 5) and protein (Fig. 6) expression levels were significantly decreased in mice treated with RPE (*P* < 0.05).

### RPE treatment increased HO-1 and Nrf2 protein expression in the lung

**Fig. 7:**
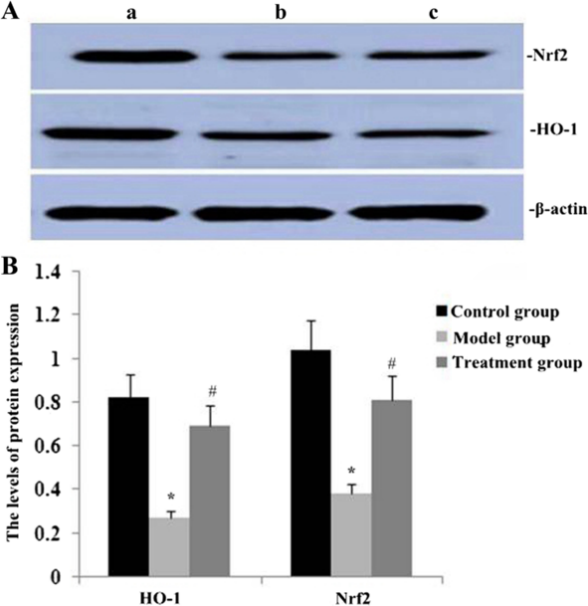
Effect of RPE treatment on protein expression of HO-1 and Nrf2 in the lung. **a** Representative western blots showing HO-1 and Nrf2 protein on day 14 after RPE treatment (1, control group; 2, model group; 3, treatment group). **b** Statistical summary of the densitometric analyses of HO-1 and Nrf2 expression. Data are means ± SD of three experiments. *P < 0.05 and **P < 0.01 vs the control group; ##*P* < 0.01 and #*P* < 0.01 vs the model group

To observe effects of RPE on expression of HO-1 and Nrf2 proteins in the lungs in PQ treated mice, we used western blotting. Protein levels of HO-1 and Nrf2 in the lung were significantly decreased during PQ-induced pulmonary fibrosisbut were significantly higher in mice also treated with RPE (Fig. 7).

### Effects of RPE treatment on FSTL1 and α-SMA staining in lungs from PQ-treated mice

**Fig. 8:**
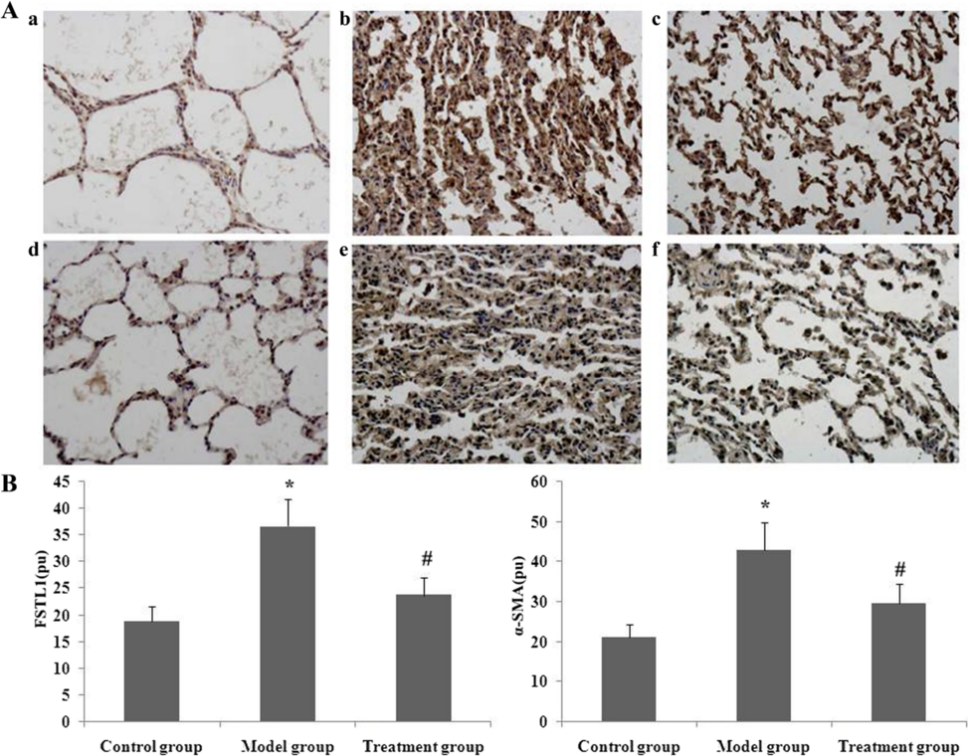
Effect of RPE treatment on the protein expression of FSTL1 and α-SMA in the lung. Immunohistochemistry was performed on lung sections after antigen retrieval with Retrievagen on day 14 after RPE treatment. **a** Representative immunostaining (brown) patterns for FSTL1 and α-SMA are shown: FSTL1 staining (a, control group; b, model group; c, treatment group); α-SMA staining (d, control group; e, model group; f, treatment group). **b** Statistical summary of the densitometric analyses of FSTL1 and α-SMA staining. Data are means ± SD. *P < 0.05 and **P < 0.01 vs the control group; ##*P* < 0.01 and #*P* < 0.01 vs the model group

Immunohistochemical analysis was used to determine distribution of FSTL1 and α-SMA in the mouse lung on day 14 after PQ or saline treatment. Staining for FSTL1 and α-SMA was localised to alveolar epithelium. The number of cells expressing FSTL1 and α-SMA was significantly increased during PQ-induced pulmonary fibrosis and this increase was significantly reduced by RPE treatment (Fig. 8).

### RPE treatment decreased TGF-β1 and MMP-9 levels in BAL from PQ-treated mice

**Fig. 9:**
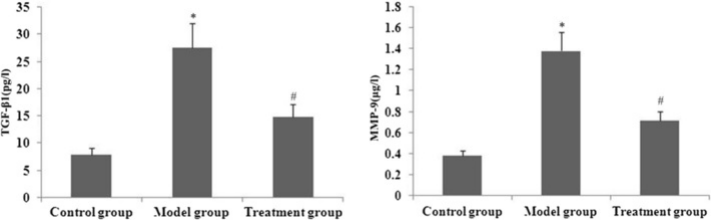
Effect of RPE treatment on levels of TGF-β1 and MMP-9 in BAL. TGF-βand MMP-9 in BALwere measured by ELISA. Data are means ± SD from triplicate experiments. **P < 0.01 and *P < 0.05 vs the control group; # *P* < 0.05 and # # *P* < 0.01 vs the model group

BAL fluids were collected 24 h after PQ treatment to evaluate levels of MMP-9 and TGF-β1. PQ caused significant pulmonary fibrosis, as indicated by increased BAL concentrations of TGF-β1 and MMP-9. Treatment of mice with RPE reduced the increases in TGF-β1 and MMP-9 levels (Fig. 9).

### RPE treatment ameliorated oxidative stress

**Fig. 10:**
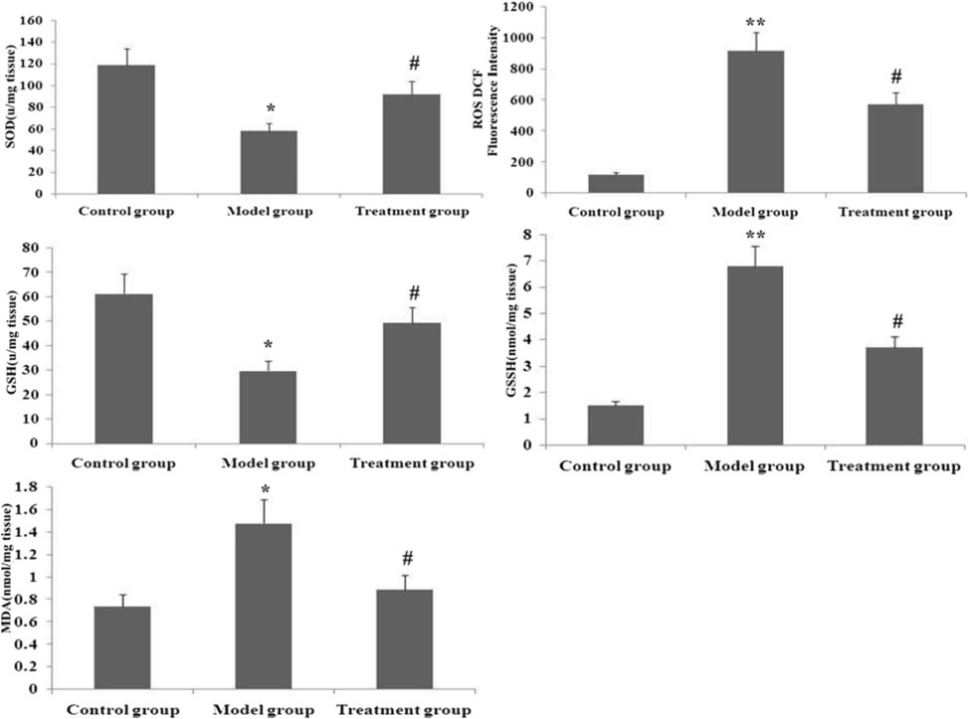
Effect of RPE treatment on SOD activity and levels of ROS, MDA, GSH and GSSG in the lung. These parameters of oxidative stress were assessed as described in Methods. Data are means ± SDas determined from three independent experiments. **P < 0.01 and *P < 0.05 vs the control group; # *P* < 0.05 vs the model group

To analyse effects of RPE on oxidative stress, SOD activity and levels of ROS, MDA, GSH and GSSG were determined. As shown in Fig. 10, ROS, MDA and GSSG levels were significantly increased and GSH levels and SOD activity were markedly decreased on day 14 after PQ administration. However, RPE treatment significantly attenuated these indicators of oxidative stress.

### RPE administration attenuated PQ-induced pulmonary fibrosis

**Fig. 11:**
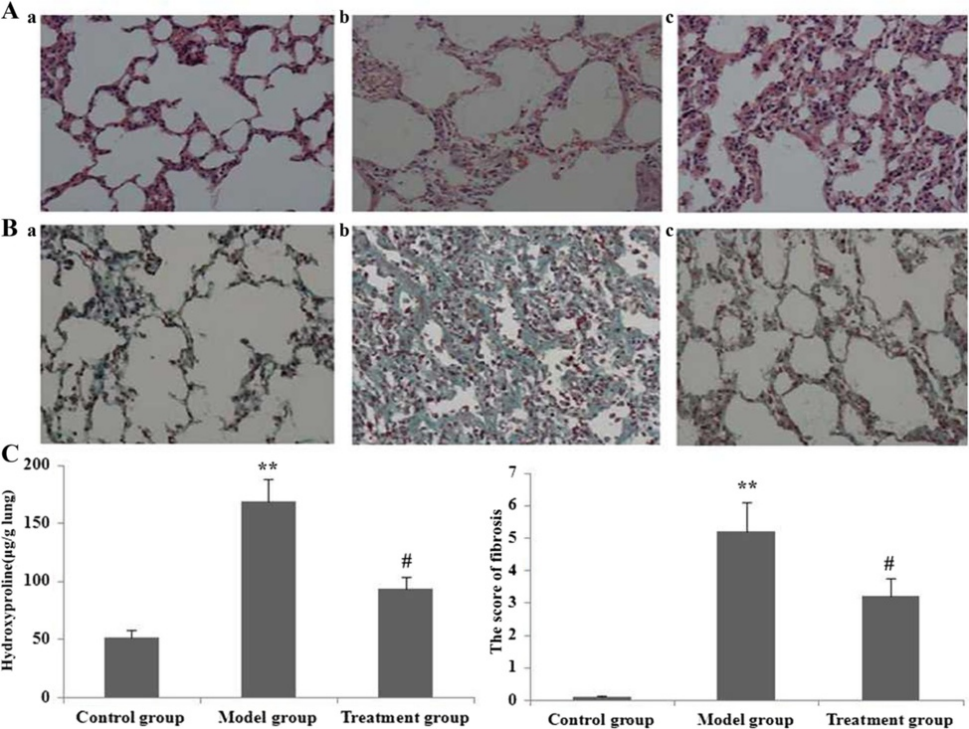
Effect of RPE treatment on lung histopathology (**a**: HE staining, 200× magnification; **b**: Masson’s trichrome staining, 200× magnification). **a** and **b** Histopathological changes in lung tissue from mice (a, control group; b, model group; c, treatment group). **c** Statistical summary of pulmonary fibrosis scores in mice. Pulmonary fibrosis scores were analyzed by nonparametric methods from triplicate experiments. **P < 0.01 vs the control group; #*P* < 0.05 vs the model group

We assessed effects of RPE treatment on PQ-induced pulmonary fibrosis using histological staining. Masson’s trichrome and H&E staining revealed significant thickening of alveolar septa and collagen deposition in the lungs on day 14 after PQ administration (Fig. 11a and b). The degree of PQ-induced pulmonary fibrosis was greatly attenuated in mice also treated with RPE.

Dense fibrosis with prominent collagen deposition was observed in the lungs on day 14 after PQ administration. The degree of dense fibrosis with collagen deposition induced by PQ was greatly attenuated by RPE treatment (Fig. 11a–c). No abnormal alveolar architecture was observed in lungs of the saline-administered control mice.

Next, we assessed the degree of pulmonary fibrosis using a scoring method. In PQ-treated mice, the scores of fibrotic lesions were significantly worse (that is, higher scores) on day 14 after PQ administration compared with those of saline-administered controls (Fig. 11c). However, the scores were significantly improved by RPE administration to PQ-treated mice.

Based on measurements of lung HYP content, we further assessed the degree of pulmonary fibrosis, observing a significant increase on day 14 after PQ administration (Fig. 11c). However, HYP content was significantly lower in PQ-treated mice that had also received RPE (Fig. 11c).

## Discussion

The herbicide PQ primarily accumulates in the lungs through the highly developed polyamine uptake system, leading to the generation of a superoxide radical (O_2_^−^) through oxygen- and NADPH-dependent redox cycling and resulting in acute oxidative stress-related insults [27, 28]. Current treatments for PQ poisoning focus on reducing its absorption from the gastrointestinal tract and on increasing its elimination [29]. Several other interventions have been proposed but none were shown to be effective in clinical trials. The most potentially promising intervention is immunosuppressive therapy but it is not widely used because it has little supporting evidence [29, 30].

Puerarin has been reported to have protective effects related to its abilities to increase SOD activity, decrease lipid peroxidation and enhance fibrinolysis [31]. Puerarin is a scavenger of oxygen-free radicals and can prophylactically reduce the oxidative injury induced by H_2_O_2_ and superoxide anion [32]. Recent studies indicated that puerarin inhibited oxidative stress induced by acute alcoholism [33], reduced PGE2, TNF and IL-6 production [34] and acted as an anti-inflammatory agent by blocking NF–kappa B signalling [34]. It was suggested that puerarin should be developed for chemoprevention of atherosclerosis [35]. In our study, RPE, extracts that contain puerarin, inhibited miR-21 expression, blocked FSTL1 expression and increased HO-1 and Nrf2 protein levels in lungs from PQ-treated mice. RPE treatment also inhibited PQ-induced effects on ROS and MDA levels, downregulated gene expression of TGF-β1, CTGF, collagen III and collagen I and ameliorated PQ-induced pulmonary fibrosis. These results support an important role of puerarin in attenuating development of pulmonary fibrosis or protecting against pulmonary fibrosis after PQ exposure.

Because miR-21 is one of the most abundant microRNAs, it plays an important role in the pathogenesis and development of fibrosis. Previous studies indicated that miR-21 was upregulated during fibrogenesis in the heart, kidney and lung [36–38]. In addition, miR-21 was significantly upregulated during liver fibrosis of different aetiologies [39], further indicating that miR-21 is a common effector in fibrotic disease. This implies that miR-21 is an attractive potential therapeutic target against fibrosis under various pathological conditions. Upregulation of miR-21 was observed in the cardiac fibroblasts of failing hearts and treatment with an miR-21 antagomir in amouse model of cardiac hypertrophy prevented interstitial fibrosis and improved cardiac function [40]. Inhibiting miR-21 expression in the lung successfully ameliorated pulmonary fibrosis and it was suggested that miR-21 promoted fibrosis by targeting the anti-fibrotic protein Smad7 [41]. Smad7, as an intrinsic inhibitor of the TGF-β/Smad2/3 pathway, competes with R-Smads for type I TGF receptors (TβRI) and intervenes in TGF-β/Smad2/3 signal transduction [41]. Previous studies indicated that adenovirus-mediated Smad7 overexpression in the mouse liver inhibited collagen deposition and α-SMA expression in hepatic stellate cells (HSCs) [42]. In our study, PQ markedly upregulated miR-21 expression, stimulated FSTL1 expression, facilitated TGF-β/Smad2/3 and p38MAPK signal transduction and induced pulmonary fibrosis. However, administration of RPE significantly suppressed PQ-induced miR-21 expression, blocked FSTL1 expression, decreased p-p38MAPK, NF-kB65, p-Smad2/3 and MMP-9 protein levels and attenuated pulmonary fibrosis.

Fstl l was upregulated in patients with IPF and in the bleomycin-induced model of pulmonary fibrosis [13]. In the lungs of bleomycin-treated WT mice, Fstl l mRNA and FSTL1 protein levels were increased and FSTL1 immunoreactivity was evident in fibrotic areas [14]. Fstl l haploinsufficiency in Fstl l +/− mice showed a clear antifibrotic effect in the lungs after bleomycin treatment, as indicated by a markedly attenuated fibrotic phenotype with significantly less collagen deposition, as compared with in wild type mice [14]. These findings confirmed the critical role of FSTL1 as a profibrotic protein during progressive interstitial fibrosis [14]. In Fst l+/− mice, insufficiency of FSTL1 attenuated bleomycin-induced fibrosis, with limited expansion of the myofibroblast pool because of impaired epithelial mesenchymal transition (EMT) and reduced Smad2/3, ERK and JNK phosphorylation [14]. Fstl l overexpression increased TGF-β1-induced EMT via Smad-dependent and MAPK-dependent pathways in A549 cells. In that study, selective chemical blockade of Smad2/3, ERK or JNK phosphorylation decreased expression of EMT marker proteins [14]. Our study demonstrated that Fstl l was upregulated through miR-21 expression after PQ challenge, resulting in increased Smad2/3 and p38MAPK phosphorylation and progressive PQ-induced pulmonary fibrosis. RPE treatment significantly downregulated Fstl l expression by decreasing miR-21 expression. This decreased the expression of profibrogenic genes and directly suppressed PQ-induced fibrosis.

Smad2/3, as the primary signalling mediator, is pivotal in the TGF-β signalling pathway. Thus, Smad2/3 mediated expression of type I collagen A1 in activated HSCs and targeted deletion of Smad2/3 prevented or halted the progression of hepatic fibrosis in animals [43]. Interestingly, in our present study, puerarin induced downregulation of Smad2/3 expression in PQ-treated mice. This likely reduced expression of profibrogenic genes and directly suppressed pulmonary fibrosis, thus protecting against miR-21-mediated injury and downregulating the major signalling transducer, Smad2/3.

CTGF is the primary downstream mediator of TGF-β-induced fibroblast activation and its specific role in fibrotic tissue makes it a better therapeutic target than TGF-β [44]. In our study, RPE extracts appeared to attenuate PQ-induced lung fibrosis through blocking TGF-β1 and CTGF expression, as a result of decreased miR-21 levels. This suggested that PQ-induced fibrosis might be mediated by miR-21-stimulated expression of the profibrotic factors TGF-β1 and CTGF.

PQ produces lung oxidative stress in animals [2] and humans [3]. Excessive levels of ROS damage cellular macromolecules, such as DNA, lipids and proteins, leading to oxidative stress-induced tissue injury [45]. Cellular antioxidants play a key role in removal or detoxification of ROS and are essential for preventing oxidative damage. HO-1 provides an inducible defence mechanism that can be activated ubiquitously in cells and tissues in response to noxious stimuli, conferring cellular protection against injury inflicted by such stimuli [46]. HO-1 may play an important role in protecting against PQ-induced tissue damage. Nrf2 is a redox-sensitive basic leucine zipper transcription factor of the NADPH oxidase complex that is activated by oxidative stress and, when activated, translocates into the nucleus [47]. Nrf2 activation has been reported to play an important role in the antioxidant response element (ARE)-driven expression of several detoxifying and antioxidant enzymes, including HO-1 [48]. In this study, puerarin increased HO-1 and Nrf2 expression, suppressed ROS and MDA levels, significantly increased SOD activity and GSH levels, decreased miR-21 and Fstl 1 expression and attenuated PQ-induced pulmonary fibrosis.

TGF-βl induces breakdown of collagen and other matrix proteins by enhancing generation of MMPs [48] and plasminogen activators and inhibiting expression of tissue inhibitors of metalloproteinases (TIMPs) [49]. Therefore, in addition to downregulating miR-21 and Fstl l expression, puerarin might interrupt the TGF-β autocrine pathway by inhibiting MMP-9 activity and, thereby, suppress PQ-induced pulmonary fibrosis.

NF-κB activation increased in an animal model of lung fibrosis and blocking NF-κB activation suppressed lung collagen deposition and inflammation, consequently exacerbating fibrosis [50]. Several lines of evidence suggest that MAPK can participate in NF-κB activation in the cytoplasm as well as the modulation of its transactivation potential in the nucleus [51]. In our experiments, RPE treatment blocked the p38MAPK pathway and inhibited NF-κB activation by suppressing Fstl 1 expression via downregulation of miR-21 expression. This reduced lung collagen deposition and alleviated PQ-induced pulmonary fibrosis.

PQ causes destruction of the lung architecture, leading to pulmonary fibrosis, which is characterised by increased HYP levels and more collagen deposition in the lungs [52]. α-SMA is a marker of fibroblast activation and its presence indicates the occurrence of the fibroblast transition towards myofibroblasts [53]. The present study showed a substantially increased intensity of lung collagen staining in PQ-treated animals, reflecting the detrimental alterations associated with fibrosis. Increased HYP levels were correlated to collagen accumulation in the alveolar space. Our data also indicated that the RPE-treated mice exhibited significantly lower HYP levels and α-SMA expression. The ameliorating effects of RPE on these histological alterations indicated suppression of Fstl l mediated effects by downregulating miR-21 expression, preventing accumulation of HYP in lungs of mice treated with PQ.

## Conclusions

The results of this study suggest a novel compensatory mechanism for the action of RPE in inhibiting lung fibrosis under its pathological induction by PQ. This mechanism involves blocking profibrotic gene and protein expression though decreasing Fstl l expression via downregulation of miR-21. We have demonstrated for the first time that RPE has protective, antifibrotic effects against pulmonary fibrosis in a PQ-induced mouse model. Thus, the beneficial effects of in vivo administration of RPE on the parameters of pulmonary fibrosis described in this study support a novel potential therapeutic modality for treating PQ-induced pulmonary fibrosis and other forms of pulmonary fibrosis.

## Abbreviations

RPE: radix puerariae extract
Fstl l: follistatin-like 1 gene
FSTL1: follistatin-like 1 protein
miR-21: microRNA-21
PQ: paraquat
CTGF: connective tissue growth factor
MMP-9: matrix metalloproteinase-9
ECM: extracellularmatrix
TIMPs: tissue inhibitors of metalloproteinases
Nrf2: nuclear factor erythroid 2p45-related factor-2
MAPKs: mitogen-activated protein kinases
NF-κB: nuclear factor kappa
α-SMA: alpha-smooth muscle actin
ROS: reactive oxygen species
HO-1: heme oxygenase-1
SOD: superoxide dismutase
TGF-β: transforming growth factor-β
MDA: malondialdehyde
GSH: reduced glutathione
GSSH: oxidised glutathione
HSCs: hepatic stellate cells
